# {4-Bromo-2-[(2-{(ethyl­sulfan­yl)[(2-oxidobenzyl­idene-κ*O*)amino-κ*N*]methylidene}hydrazinyl­idene-κ*N* ^1^)meth­yl]phenolato-κ*O*}(butan-2-ol-κ*O*)dioxidouranium(VI)

**DOI:** 10.1107/S1600536812005077

**Published:** 2012-02-10

**Authors:** Reza Takjoo, Mehdi Ahmadi, Seik Weng Ng, Edward R. T. Tiekink

**Affiliations:** a Department of Chemistry, School of Sciences, Ferdowsi University of Mashhad, 91775-1436 Mashhad, Iran; b Department of Chemistry, University of Malaya, 50603 Kuala Lumpur, Malaysia; c Chemistry Department, Faculty of Science, King Abdulaziz University, PO Box 80203 Jeddah, Saudi Arabia

## Abstract

The U^VI^ cation in the title complex, [U(C_17_H_14_BrN_3_O_2_S)O_2_(C_4_H_10_O)], exists within a distorted penta­gonal–bipyramidal geometry, where the oxide atoms occupy the axial positions [O—U—O = 179.8 (3)°] and the penta­gonal plane is defined by the N_2_O_2_ atoms of the tetra­dentate Schiff base ligand and the O atom of the 2-butanol mol­ecule. In the crystal, centrosymmetric aggregates are formed *via* pairs of hy­droxy–phenolate O—H⋯O hydrogen bonds. The azomethine C=N atoms, the ethyl­thiolyl group, the 2-butanol mol­ecule and Br atom are disordered over two positions in a 0.627 (3):0.373 (3) ratio.

## Related literature

For background to uranyl Schiff base complexes, see: Şahin *et al.* (2010 ▶); Özdemir *et al.* (2011 ▶). For a related structure, see: Takjoo *et al.* (2012 ▶).
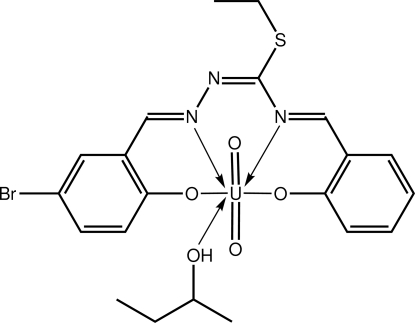


## Experimental

### 

#### Crystal data

[U(C_17_H_14_BrN_3_O_2_)O_2_(C_4_H_10_O)]
*M* *_r_* = 748.43
Monoclinic, 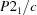

*a* = 11.4795 (2) Å
*b* = 14.6450 (3) Å
*c* = 14.3383 (6) Å
β = 98.094 (3)°
*V* = 2386.50 (12) Å^3^
*Z* = 4
Mo *K*α radiation
μ = 8.60 mm^−1^
*T* = 100 K
0.30 × 0.10 × 0.05 mm

#### Data collection

Agilent SuperNova Dual diffractometer with Atlas detector
Absorption correction: multi-scan (*CrysAlis PRO*; Agilent, 2010 ▶) *T*_min_ = 0.182, *T*_max_ = 0.673
17329 measured reflections
5495 independent reflections
4365 reflections with *I* > 2σ(*I*)
*R*_int_ = 0.046

#### Refinement

*R*[*F* ^2^ > 2σ(*F* ^2^)] = 0.052
*wR*(*F* ^2^) = 0.102
*S* = 1.18
5495 reflections
326 parameters
44 restraints
H-atom parameters constrained
Δρ_max_ = 1.58 e Å^−3^
Δρ_min_ = −0.97 e Å^−3^

### 

Data collection: *CrysAlis PRO* (Agilent, 2010 ▶); cell refinement: *CrysAlis PRO*; data reduction: *CrysAlis PRO*; program(s) used to solve structure: *SHELXS97* (Sheldrick, 2008 ▶); program(s) used to refine structure: *SHELXL97* (Sheldrick, 2008 ▶); molecular graphics: *X-SEED* (Barbour, 2001 ▶) and *DIAMOND* (Brandenburg, 2006 ▶); software used to prepare material for publication: *publCIF* (Westrip, 2010 ▶).

## Supplementary Material

Crystal structure: contains datablock(s) global, I. DOI: 10.1107/S1600536812005077/hb6627sup1.cif

Structure factors: contains datablock(s) I. DOI: 10.1107/S1600536812005077/hb6627Isup2.hkl

Additional supplementary materials:  crystallographic information; 3D view; checkCIF report

## Figures and Tables

**Table 1:** Selected bond lengths (Å)

U—O1	2.297 (6)
U—O2	2.232 (6)
U—O3	1.777 (5)
U—O4	1.777 (5)
U—O5	2.411 (5)
U—N1	2.572 (7)
U—N3	2.563 (6)

**Table 2:** Hydrogen-bond geometry (Å, °)

*D*—H⋯*A*	*D*—H	H⋯*A*	*D*⋯*A*	*D*—H⋯*A*
O5—H5O⋯O1^i^	0.84	1.88	2.667 (8)	155

Symmetry code: (i) 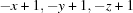.

**Fig. 1.**
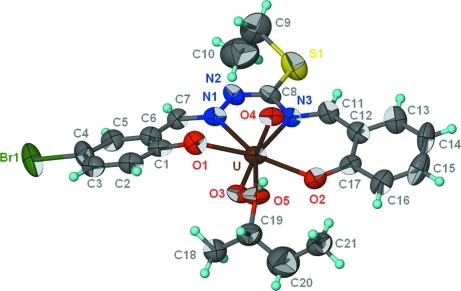
The molecular structure of (I) showing displacement ellipsoids at the 70% probability level. Only the major component of the disordered residues are shown.

**Fig. 2.**
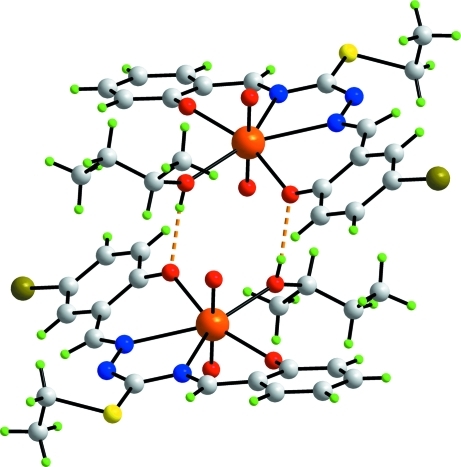
A view of the centrosymmetric aggregate in (I). The O—H···O hydrogen bonds are shown as dashed lines.

**Fig. 3.**
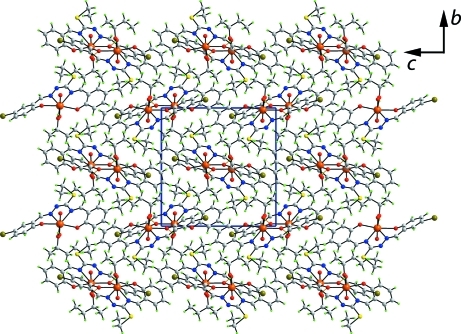
A view in projection down the a axis of the unit-cell contents of (I).

## supplementary crystallographic information

### Comment

Recent studies of uranyl Schiff base complexes (Şahin *et al.*, 2010) motivated the synthesis of the title complex, (I), in continuation of related structural studies (Takjoo *et al.*, 2012).

The U atom in (I), Fig. 1, exists within a distorted pentagonal bipyramidal geometry with the axial positions occupied by the oxido-O atoms, O3—U—O4 = 179.8 (3)°. The pentagonal plane is defined by the N_2_O_2_ atoms, derived from the tetradentate Schiff base ligand, and the O atom of the 2-butanol molecule, Table 1. The Schiff base ligand is somewhat twisted with the dihedral angle between the terminal benzene rings being 31.9 (4)°.

In the crystal structure, centrosymmetric pairs of molecules are linked *via* O—H···O hydrogen bonds formed between the hydroxyl and O1-phenoxide atoms, Fig. 2 and Table 2. The dimeric aggregates stack into columns parallel to *a*, Fig. 3.

### Experimental

UO_2_(OAc)_2_.2H_2_O (0.42 g, 1.0 mmol) was added to a butanol solution (20 ml) of 5-bromosalicylaldehyde mono-*S*-ethylisothiosemicarbazone hydrobromide (0.38 g, 1.0 mmol) and salicylaldehyde (0.12 g, 1.0 mmol). The red solution was heated under reflux for 1 h at 70 °C. Red prisms were isolated after four days, collected by filtration, washed with diethyl ether and dried in air. m.p. 493 K (dec,). Yield: 33%.

### Refinement

Carbon-bound H atoms were placed in calculated positions [O—H = 0.84 Å and C—H 0.95 to 0.99 Å, *U*_iso_(H) = 1.2 to 1.5*U*_eq_(O,C)] and were included in the refinement in the riding model approximation.

The ethylthiolyl unit is disordered over two positions; the minor component refined to a site occupancy of 0.373 (3). The isotropic displacement parameters of the atoms of the minor component were constrained to be equal to *U*_eq_ of the major component. Pairs of S—C and C—C distances were restrained to within 0.01 Å of each other. The azomethine C═N unit is also disordered; the positions and anisotropic displacement parameters of the primed atoms were set to those of the unprimed ones. The bromine substituent is also similarly disordered over two benzene rings.

The 2-butanol molecule is also disordered over two positions, and the occupancies were set to those of the ethylthiolyl unit. The isotropic displacement parameters of the atoms of the minor component were constrained to be equal to *U*_eq_ of the major component; the C19, C20 and C21 atoms were restrained to be nearly isotropic. Pairs of C—O and C—C distances were restrained to within 0.01 Å of each other. The C—C distances were restrained to 1.54±0.01 Å and the non-bonded C···C distances to 2.51±0.01 Å.

The final difference Fourier map had a peak at 0.24 Å from H4 and a hole at 0.66 Å from U.

### Crystal data

**Table:**

[U(C_17_H_14_BrN_3_O_2_)O_2_(C_4_H_10_O)]	*F*(000) = 1416
*M**_r_* = 748.43	*D*_x_ = 2.083 Mg m^−^^3^
Monoclinic, *P*2_1_/*c*	Mo *K*α radiation, λ = 0.71073 Å
Hall symbol: -P 2ybc	Cell parameters from 5878 reflections
*a* = 11.4795 (2) Å	θ = 2.3–27.5°
*b* = 14.6450 (3) Å	µ = 8.60 mm^−^^1^
*c* = 14.3383 (6) Å	*T* = 100 K
β = 98.094 (3)°	Prism, red
*V* = 2386.50 (12) Å^3^	0.30 × 0.10 × 0.05 mm
*Z* = 4	

### Data collection

**Table:**

Agilent SuperNova Dual diffractometer with Atlas detector	5495 independent reflections
Radiation source: SuperNova (Mo) X-ray Source	4365 reflections with *I* > 2σ(*I*)
Mirror monochromator	*R*_int_ = 0.046
Detector resolution: 10.4041 pixels mm^-1^	θ_max_ = 27.6°, θ_min_ = 2.3°
ω scans	*h* = −14→14
Absorption correction: multi-scan (*CrysAlis PRO*; Agilent, 2010)	*k* = −19→19
*T*_min_ = 0.182, *T*_max_ = 0.673	*l* = −14→18
17329 measured reflections	

### Refinement

**Table:**

Refinement on *F*^2^	Primary atom site location: structure-invariant direct methods
Least-squares matrix: full	Secondary atom site location: difference Fourier map
*R*[*F*^2^ > 2σ(*F*^2^)] = 0.052	Hydrogen site location: inferred from neighbouring sites
*wR*(*F*^2^) = 0.102	H-atom parameters constrained
*S* = 1.18	*w* = 1/[σ^2^(*F*_o_^2^) + (0.0173*P*)^2^ + 17.3449*P*] where *P* = (*F*_o_^2^ + 2*F*_c_^2^)/3
5495 reflections	(Δ/σ)_max_ = 0.001
326 parameters	Δρ_max_ = 1.58 e Å^−^^3^
44 restraints	Δρ_min_ = −0.97 e Å^−^^3^

### Fractional atomic coordinates and isotropic or equivalent isotropic displacement parameters (Å^2^)

**Table:**

	*x*	*y*	*z*	*U*_iso_*/*U*_eq_	Occ. (<1)
U	0.71638 (2)	0.51761 (2)	0.61360 (2)	0.03255 (10)	
Br1	0.89529 (14)	0.43057 (14)	0.11495 (12)	0.0647 (6)	0.627 (2)
Br1'	0.6959 (2)	0.67063 (17)	1.12727 (18)	0.0459 (8)	0.373 (2)
S1	1.0253 (4)	0.7635 (3)	0.7352 (4)	0.0648 (13)	0.627 (2)
S1'	1.0601 (4)	0.7180 (3)	0.5330 (4)	0.0285 (12)	0.373 (2)
O1	0.6612 (4)	0.5191 (4)	0.4533 (4)	0.0417 (14)	
O2	0.7143 (5)	0.4871 (4)	0.7659 (4)	0.0395 (14)	
O3	0.8190 (4)	0.4273 (3)	0.6093 (4)	0.0366 (14)	
O4	0.6138 (4)	0.6079 (4)	0.6175 (4)	0.0364 (14)	
O5	0.5534 (5)	0.4123 (4)	0.5981 (5)	0.0418 (15)	0.627 (2)
H5O	0.4914	0.4432	0.5965	0.063*	0.627 (2)
O5'	0.5534 (5)	0.4123 (4)	0.5981 (5)	0.0418 (15)	0.373
H5O'	0.4859	0.4304	0.5753	0.063*	0.627 (2)
N1	0.8701 (5)	0.6029 (4)	0.5323 (5)	0.0343 (16)	
N2	0.9460 (6)	0.6668 (5)	0.5850 (5)	0.0363 (18)	0.627 (2)
C8'	0.9460 (6)	0.6668 (5)	0.5850 (5)	0.0363 (18)	0.373
N3	0.8540 (5)	0.6299 (4)	0.7120 (5)	0.0313 (15)	
C1	0.7170 (7)	0.4970 (5)	0.3816 (6)	0.035 (2)	
C2	0.6604 (7)	0.4462 (6)	0.3064 (7)	0.044 (2)	
H2	0.5829	0.4243	0.3084	0.052*	
C3	0.7155 (8)	0.4273 (6)	0.2288 (7)	0.045 (2)	
H3	0.6757	0.3928	0.1779	0.054*	
C4	0.8294 (8)	0.4590 (7)	0.2250 (7)	0.053 (3)	
H4	0.8670	0.4466	0.1714	0.063*	0.373 (2)
C5	0.8863 (7)	0.5075 (6)	0.2985 (6)	0.042 (2)	
H5	0.9641	0.5281	0.2955	0.050*	
C6	0.8338 (6)	0.5282 (5)	0.3788 (6)	0.0344 (18)	
C7	0.8987 (7)	0.5843 (5)	0.4495 (6)	0.0344 (19)	
H7	0.9695	0.6106	0.4347	0.041*	
C8	0.9367 (6)	0.6810 (5)	0.6703 (6)	0.0336 (18)	0.627 (2)
N2'	0.9367 (6)	0.6810 (5)	0.6703 (6)	0.0336 (18)	0.373
C9	1.0718 (19)	0.8414 (15)	0.6368 (19)	0.080 (6)	0.627 (2)
H9A	1.0115	0.8464	0.5804	0.097*	0.627 (2)
H9B	1.1000	0.9025	0.6594	0.097*	0.627 (2)
C10	1.160 (2)	0.7813 (18)	0.626 (2)	0.114 (9)	0.627 (2)
H10A	1.2025	0.8020	0.5755	0.171*	0.627 (2)
H10B	1.1268	0.7207	0.6111	0.171*	0.627 (2)
H10C	1.2151	0.7780	0.6854	0.171*	0.627 (2)
C9'	1.116 (3)	0.800 (2)	0.636 (3)	0.080 (6)	0.373
H9'A	1.0956	0.7773	0.6966	0.097*	0.373 (2)
H9'B	1.2028	0.8082	0.6420	0.097*	0.373 (2)
C10'	1.059 (4)	0.881 (3)	0.610 (4)	0.114 (9)	0.373
H10D	1.0978	0.9311	0.6466	0.171*	0.373 (2)
H10E	0.9769	0.8762	0.6223	0.171*	0.373 (2)
H10F	1.0598	0.8915	0.5428	0.171*	0.373 (2)
C11	0.8440 (7)	0.6554 (6)	0.7987 (6)	0.038 (2)	
H11	0.8901	0.7066	0.8219	0.046*	
C12	0.7717 (7)	0.6153 (6)	0.8632 (6)	0.039 (2)	
C13	0.7703 (8)	0.6596 (7)	0.9479 (7)	0.051 (2)	
H13	0.8141	0.7144	0.9605	0.061*	
C14	0.7061 (8)	0.6254 (8)	1.0150 (7)	0.053 (3)	
H14	0.7045	0.6568	1.0728	0.064*	0.373 (2)
C15	0.6442 (8)	0.5449 (8)	0.9963 (7)	0.058 (3)	
H15	0.6004	0.5208	1.0421	0.069*	
C16	0.6451 (7)	0.4991 (7)	0.9129 (7)	0.047 (2)	
H16	0.6016	0.4441	0.9015	0.056*	
C17	0.7098 (6)	0.5329 (5)	0.8440 (6)	0.0345 (19)	
C18	0.6029 (13)	0.2716 (10)	0.5306 (11)	0.057 (4)	0.627 (2)
H18A	0.6122	0.3110	0.4769	0.085*	0.627 (2)
H18B	0.5703	0.2126	0.5075	0.085*	0.627 (2)
H18C	0.6797	0.2621	0.5687	0.085*	0.627 (2)
C19	0.5196 (13)	0.3168 (7)	0.5904 (9)	0.056 (4)	0.627 (2)
H19	0.4394	0.3146	0.5528	0.067*	0.627 (2)
C20	0.512 (2)	0.2656 (13)	0.6807 (13)	0.100 (7)	0.627 (2)
H20A	0.5924	0.2567	0.7149	0.120*	0.627 (2)
H20B	0.4775	0.2045	0.6654	0.120*	0.627 (2)
C21	0.4374 (14)	0.3160 (11)	0.7449 (10)	0.062 (4)	0.627 (2)
H21A	0.4268	0.2770	0.7986	0.093*	0.627 (2)
H21B	0.3604	0.3306	0.7093	0.093*	0.627 (2)
H21C	0.4772	0.3725	0.7677	0.093*	0.627 (2)
C18'	0.484 (2)	0.2953 (18)	0.6972 (18)	0.057 (4)	0.373
H18D	0.4965	0.3373	0.7510	0.085*	0.373 (2)
H18E	0.4957	0.2324	0.7196	0.085*	0.373 (2)
H18F	0.4028	0.3023	0.6652	0.085*	0.373 (2)
C19'	0.5709 (19)	0.3176 (9)	0.6280 (14)	0.056 (4)	0.373
H19'	0.6532	0.3094	0.6604	0.067*	0.373 (2)
C20'	0.548 (3)	0.252 (2)	0.544 (2)	0.100 (7)	0.373
H20C	0.5547	0.1880	0.5668	0.120*	0.373 (2)
H20D	0.4678	0.2613	0.5104	0.120*	0.373 (2)
C21'	0.638 (2)	0.269 (2)	0.4770 (17)	0.062 (4)	0.373
H21D	0.6245	0.2264	0.4239	0.093*	0.373 (2)
H21E	0.7179	0.2598	0.5107	0.093*	0.373 (2)
H21F	0.6304	0.3318	0.4534	0.093*	0.373 (2)

### Atomic displacement parameters (Å^2^)

**Table:**

	*U*^11^	*U*^22^	*U*^33^	*U*^12^	*U*^13^	*U*^23^
U	0.01734 (14)	0.03167 (16)	0.0468 (2)	−0.00210 (12)	−0.00192 (11)	−0.00001 (15)
Br1	0.0434 (9)	0.1021 (14)	0.0438 (10)	0.0328 (9)	−0.0099 (7)	−0.0256 (9)
Br1'	0.0460 (15)	0.0473 (15)	0.0454 (16)	−0.0007 (11)	0.0102 (11)	−0.0049 (11)
S1	0.049 (2)	0.067 (3)	0.082 (4)	−0.024 (2)	0.020 (2)	−0.025 (2)
S1'	0.022 (2)	0.035 (3)	0.031 (3)	−0.016 (2)	0.012 (2)	−0.010 (2)
O1	0.022 (3)	0.052 (4)	0.048 (4)	−0.005 (3)	−0.003 (3)	−0.005 (3)
O2	0.029 (3)	0.043 (3)	0.046 (4)	−0.005 (3)	0.002 (3)	0.003 (3)
O3	0.022 (3)	0.030 (3)	0.058 (4)	0.004 (2)	0.006 (3)	−0.003 (3)
O4	0.017 (2)	0.033 (3)	0.059 (4)	0.001 (2)	0.002 (2)	0.004 (3)
O5	0.026 (3)	0.030 (3)	0.068 (5)	−0.006 (2)	0.001 (3)	0.005 (3)
O5'	0.026 (3)	0.030 (3)	0.068 (5)	−0.006 (2)	0.001 (3)	0.005 (3)
N1	0.022 (3)	0.032 (4)	0.045 (5)	−0.007 (3)	−0.005 (3)	0.000 (3)
N2	0.025 (4)	0.036 (4)	0.047 (5)	−0.007 (3)	0.005 (3)	−0.010 (3)
C8'	0.025 (4)	0.036 (4)	0.047 (5)	−0.007 (3)	0.005 (3)	−0.010 (3)
N3	0.019 (3)	0.035 (4)	0.038 (4)	−0.004 (3)	−0.003 (3)	−0.003 (3)
C1	0.024 (4)	0.029 (5)	0.049 (5)	−0.002 (3)	−0.007 (4)	0.005 (4)
C2	0.031 (4)	0.043 (5)	0.054 (6)	−0.002 (4)	−0.006 (4)	0.001 (4)
C3	0.043 (5)	0.043 (5)	0.044 (6)	−0.001 (4)	−0.008 (4)	−0.001 (4)
C4	0.050 (6)	0.067 (7)	0.038 (6)	0.013 (5)	−0.007 (4)	−0.015 (5)
C5	0.029 (4)	0.048 (5)	0.046 (5)	0.006 (4)	−0.003 (4)	0.005 (4)
C6	0.022 (4)	0.037 (5)	0.041 (5)	−0.001 (3)	−0.003 (3)	−0.001 (4)
C7	0.021 (4)	0.042 (5)	0.038 (5)	−0.002 (3)	−0.002 (3)	0.002 (4)
C8	0.025 (4)	0.029 (4)	0.046 (5)	−0.010 (3)	0.002 (3)	−0.010 (3)
N2'	0.025 (4)	0.029 (4)	0.046 (5)	−0.010 (3)	0.002 (3)	−0.010 (3)
C9	0.061 (9)	0.090 (10)	0.091 (9)	−0.046 (8)	0.010 (7)	0.001 (8)
C10	0.116 (12)	0.091 (11)	0.128 (12)	−0.017 (9)	−0.011 (9)	0.009 (8)
C9'	0.061 (9)	0.090 (10)	0.091 (9)	−0.046 (8)	0.010 (7)	0.001 (8)
C10'	0.116 (12)	0.091 (11)	0.128 (12)	−0.017 (9)	−0.011 (9)	0.009 (8)
C11	0.023 (4)	0.047 (5)	0.043 (6)	−0.005 (4)	0.001 (4)	−0.006 (4)
C12	0.023 (4)	0.044 (5)	0.046 (6)	−0.001 (4)	−0.005 (4)	−0.004 (4)
C13	0.031 (5)	0.070 (7)	0.051 (7)	−0.005 (4)	0.003 (4)	−0.008 (5)
C14	0.038 (5)	0.092 (8)	0.032 (6)	−0.001 (5)	0.010 (4)	−0.001 (5)
C15	0.028 (5)	0.103 (9)	0.041 (6)	−0.007 (5)	0.004 (4)	0.019 (6)
C16	0.028 (4)	0.066 (7)	0.044 (6)	−0.001 (4)	−0.002 (4)	0.011 (5)
C17	0.020 (4)	0.040 (5)	0.043 (5)	0.005 (3)	0.000 (3)	0.006 (4)
C18	0.046 (9)	0.033 (7)	0.089 (13)	−0.008 (6)	0.005 (8)	0.027 (8)
C19	0.061 (8)	0.047 (6)	0.057 (8)	−0.005 (6)	0.000 (6)	0.003 (6)
C20	0.102 (11)	0.091 (10)	0.108 (11)	0.013 (8)	0.016 (8)	0.005 (8)
C21	0.061 (7)	0.062 (7)	0.063 (8)	−0.009 (6)	0.009 (6)	0.012 (6)
C18'	0.046 (9)	0.033 (7)	0.089 (13)	−0.008 (6)	0.005 (8)	0.027 (8)
C19'	0.061 (8)	0.047 (6)	0.057 (8)	−0.005 (6)	0.000 (6)	0.003 (6)
C20'	0.102 (11)	0.091 (10)	0.108 (11)	0.013 (8)	0.016 (8)	0.005 (8)
C21'	0.061 (7)	0.062 (7)	0.063 (8)	−0.009 (6)	0.009 (6)	0.012 (6)

### Geometric parameters (Å, º)

**Table:**

U—O1	2.297 (6)	C9'—H9'B	0.9900
U—O2	2.232 (6)	C10'—H10D	0.9800
U—O3	1.777 (5)	C10'—H10E	0.9800
U—O4	1.777 (5)	C10'—H10F	0.9800
U—O5	2.411 (5)	C11—C12	1.451 (12)
U—N1	2.572 (7)	C11—H11	0.9500
U—N3	2.563 (6)	C12—C13	1.380 (13)
Br1—C4	1.889 (10)	C12—C17	1.408 (11)
Br1'—C14	1.760 (10)	C13—C14	1.384 (13)
S1—C8	1.759 (7)	C13—H13	0.9500
S1—C9	1.95 (2)	C14—C15	1.383 (14)
S1'—C9'	1.94 (2)	C14—H14	0.9500
O1—C1	1.324 (10)	C15—C16	1.373 (14)
O2—C17	1.313 (10)	C15—H15	0.9500
O5—C19	1.452 (12)	C16—C17	1.406 (12)
O5—H5O	0.8400	C16—H16	0.9500
N1—C7	1.304 (10)	C18—C19	1.522 (9)
N1—N2	1.421 (9)	C18—H18A	0.9800
N2—C8	1.261 (10)	C18—H18B	0.9800
N3—C11	1.317 (10)	C18—H18C	0.9800
N3—C8	1.406 (9)	C19—C20	1.510 (9)
C1—C2	1.394 (12)	C19—H19	1.0000
C1—C6	1.423 (10)	C20—C21	1.532 (9)
C2—C3	1.383 (13)	C20—H20A	0.9900
C2—H2	0.9500	C20—H20B	0.9900
C3—C4	1.396 (13)	C21—H21A	0.9800
C3—H3	0.9500	C21—H21B	0.9800
C4—C5	1.359 (12)	C21—H21C	0.9800
C4—H4	0.9500	C18'—C19'	1.542 (10)
C5—C6	1.406 (12)	C18'—H18D	0.9800
C5—H5	0.9500	C18'—H18E	0.9800
C6—C7	1.430 (11)	C18'—H18F	0.9800
C7—H7	0.9500	C19'—C20'	1.535 (10)
C9—C10	1.37 (3)	C19'—H19'	1.0000
C9—H9A	0.9900	C20'—C21'	1.528 (10)
C9—H9B	0.9900	C20'—H20C	0.9900
C10—H10A	0.9800	C20'—H20D	0.9900
C10—H10B	0.9800	C21'—H21D	0.9800
C10—H10C	0.9800	C21'—H21E	0.9800
C9'—C10'	1.38 (3)	C21'—H21F	0.9800
C9'—H9'A	0.9900		
			
O3—U—O4	179.8 (3)	S1'—C9'—H9'B	111.1
O3—U—O2	89.0 (2)	H9'A—C9'—H9'B	109.1
O4—U—O2	91.1 (2)	C9'—C10'—H10D	109.5
O3—U—O1	93.6 (2)	C9'—C10'—H10E	109.5
O4—U—O1	86.2 (2)	H10D—C10'—H10E	109.5
O2—U—O1	160.29 (19)	C9'—C10'—H10F	109.5
O3—U—O5	91.8 (2)	H10D—C10'—H10F	109.5
O4—U—O5	88.3 (2)	H10E—C10'—H10F	109.5
O2—U—O5	81.2 (2)	N3—C11—C12	128.3 (8)
O1—U—O5	79.2 (2)	N3—C11—H11	115.8
O3—U—N3	97.8 (2)	C12—C11—H11	115.8
O4—U—N3	82.2 (2)	C13—C12—C17	120.5 (9)
O2—U—N3	71.4 (2)	C13—C12—C11	116.7 (8)
O1—U—N3	127.4 (2)	C17—C12—C11	122.7 (8)
O5—U—N3	150.7 (2)	C12—C13—C14	121.0 (10)
O3—U—N1	81.1 (2)	C12—C13—H13	119.5
O4—U—N1	98.8 (2)	C14—C13—H13	119.5
O2—U—N1	130.0 (2)	C15—C14—C13	118.9 (10)
O1—U—N1	69.6 (2)	C15—C14—Br1'	113.5 (8)
O5—U—N1	147.4 (2)	C13—C14—Br1'	127.6 (9)
N3—U—N1	61.9 (2)	C15—C14—H14	120.6
C8—S1—C9	102.5 (8)	C13—C14—H14	120.6
C1—O1—U	132.8 (5)	C16—C15—C14	121.2 (9)
C17—O2—U	137.6 (5)	C16—C15—H15	119.4
C19—O5—U	145.0 (7)	C14—C15—H15	119.4
C19—O5—H5O	107.5	C15—C16—C17	120.7 (9)
U—O5—H5O	107.5	C15—C16—H16	119.7
C7—N1—N2	114.0 (6)	C17—C16—H16	119.7
C7—N1—U	126.0 (5)	O2—C17—C16	120.5 (8)
N2—N1—U	119.2 (5)	O2—C17—C12	121.7 (8)
C8—N2—N1	119.7 (6)	C16—C17—C12	117.8 (9)
C11—N3—C8	113.8 (6)	C19—C18—H18A	109.5
C11—N3—U	124.9 (5)	C19—C18—H18B	109.5
C8—N3—U	120.5 (5)	H18A—C18—H18B	109.5
O1—C1—C2	120.5 (7)	C19—C18—H18C	109.5
O1—C1—C6	120.2 (7)	H18A—C18—H18C	109.5
C2—C1—C6	119.2 (8)	H18B—C18—H18C	109.5
C3—C2—C1	120.9 (8)	O5—C19—C20	117.4 (13)
C3—C2—H2	119.6	O5—C19—C18	106.2 (10)
C1—C2—H2	119.6	C20—C19—C18	112.6 (8)
C2—C3—C4	120.1 (8)	O5—C19—H19	106.7
C2—C3—H3	119.9	C20—C19—H19	106.7
C4—C3—H3	119.9	C18—C19—H19	106.7
C5—C4—C3	119.6 (9)	C19—C20—C21	112.4 (9)
C5—C4—Br1	123.9 (8)	C19—C20—H20A	109.1
C3—C4—Br1	116.5 (7)	C21—C20—H20A	109.1
C5—C4—H4	120.2	C19—C20—H20B	109.1
C3—C4—H4	120.2	C21—C20—H20B	109.1
C4—C5—C6	122.3 (8)	H20A—C20—H20B	107.9
C4—C5—H5	118.9	C20—C21—H21A	109.5
C6—C5—H5	118.9	C20—C21—H21B	109.5
C5—C6—C1	117.9 (8)	H21A—C21—H21B	109.5
C5—C6—C7	117.6 (7)	C20—C21—H21C	109.5
C1—C6—C7	124.3 (8)	H21A—C21—H21C	109.5
N1—C7—C6	126.3 (7)	H21B—C21—H21C	109.5
N1—C7—H7	116.8	C19'—C18'—H18D	109.5
C6—C7—H7	116.8	C19'—C18'—H18E	109.5
N2—C8—N3	118.7 (6)	H18D—C18'—H18E	109.5
N2—C8—S1	120.3 (6)	C19'—C18'—H18F	109.5
N3—C8—S1	121.0 (6)	H18D—C18'—H18F	109.5
C10—C9—S1	89.2 (18)	H18E—C18'—H18F	109.5
C10—C9—H9A	113.8	C20'—C19'—C18'	108.6 (9)
S1—C9—H9A	113.8	C20'—C19'—H19'	109.5
C10—C9—H9B	113.8	C18'—C19'—H19'	109.5
S1—C9—H9B	113.8	C21'—C20'—C19'	109.6 (10)
H9A—C9—H9B	111.0	C21'—C20'—H20C	109.7
C9—C10—H10A	109.5	C19'—C20'—H20C	109.7
C9—C10—H10B	109.5	C21'—C20'—H20D	109.7
H10A—C10—H10B	109.5	C19'—C20'—H20D	109.7
C9—C10—H10C	109.5	H20C—C20'—H20D	108.2
H10A—C10—H10C	109.5	C20'—C21'—H21D	109.5
H10B—C10—H10C	109.5	C20'—C21'—H21E	109.5
C10'—C9'—S1'	103 (3)	H21D—C21'—H21E	109.5
C10'—C9'—H9'A	111.1	C20'—C21'—H21F	109.5
S1'—C9'—H9'A	111.1	H21D—C21'—H21F	109.5
C10'—C9'—H9'B	111.1	H21E—C21'—H21F	109.5
			
O3—U—O1—C1	−28.2 (7)	C1—C2—C3—C4	0.2 (13)
O4—U—O1—C1	151.8 (7)	C2—C3—C4—C5	0.7 (14)
O2—U—O1—C1	−125.4 (7)	C2—C3—C4—Br1	−179.2 (7)
O5—U—O1—C1	−119.3 (7)	C3—C4—C5—C6	−0.7 (14)
N3—U—O1—C1	74.5 (7)	Br1—C4—C5—C6	179.2 (7)
N1—U—O1—C1	50.9 (6)	C4—C5—C6—C1	−0.3 (13)
O3—U—O2—C17	141.6 (7)	C4—C5—C6—C7	−176.3 (8)
O4—U—O2—C17	−38.4 (7)	O1—C1—C6—C5	−176.2 (7)
O1—U—O2—C17	−120.3 (8)	C2—C1—C6—C5	1.1 (11)
O5—U—O2—C17	−126.4 (7)	O1—C1—C6—C7	−0.4 (12)
N3—U—O2—C17	43.0 (7)	C2—C1—C6—C7	176.9 (8)
N1—U—O2—C17	64.1 (8)	N2—N1—C7—C6	−175.0 (7)
O3—U—O5—C19	2.1 (11)	U—N1—C7—C6	15.4 (11)
O4—U—O5—C19	−178.1 (11)	C5—C6—C7—N1	−172.5 (8)
O2—U—O5—C19	−86.7 (11)	C1—C6—C7—N1	11.7 (13)
O1—U—O5—C19	95.4 (11)	N1—N2—C8—N3	2.6 (11)
N3—U—O5—C19	−107.4 (11)	N1—N2—C8—S1	−177.1 (6)
N1—U—O5—C19	78.2 (12)	C11—N3—C8—N2	−174.4 (7)
O3—U—N1—C7	64.1 (6)	U—N3—C8—N2	−3.8 (9)
O4—U—N1—C7	−115.8 (6)	C11—N3—C8—S1	5.3 (9)
O2—U—N1—C7	145.3 (6)	U—N3—C8—S1	175.9 (4)
O1—U—N1—C7	−33.1 (6)	C9—S1—C8—N2	21.2 (11)
O5—U—N1—C7	−15.1 (8)	C9—S1—C8—N3	−158.5 (9)
N3—U—N1—C7	168.0 (7)	C8—S1—C9—C10	−83.3 (15)
O3—U—N1—N2	−105.0 (5)	C8—N3—C11—C12	−177.0 (8)
O4—U—N1—N2	75.1 (5)	U—N3—C11—C12	12.8 (12)
O2—U—N1—N2	−23.8 (6)	N3—C11—C12—C13	−175.1 (8)
O1—U—N1—N2	157.8 (6)	N3—C11—C12—C17	8.0 (13)
O5—U—N1—N2	175.8 (4)	C17—C12—C13—C14	−1.6 (13)
N3—U—N1—N2	−1.1 (5)	C11—C12—C13—C14	−178.5 (8)
C7—N1—N2—C8	−170.7 (7)	C12—C13—C14—C15	1.0 (14)
U—N1—N2—C8	−0.3 (9)	C12—C13—C14—Br1'	178.6 (7)
O3—U—N3—C11	−112.5 (7)	C13—C14—C15—C16	−0.4 (15)
O4—U—N3—C11	67.6 (7)	Br1'—C14—C15—C16	−178.3 (7)
O2—U—N3—C11	−26.2 (6)	C14—C15—C16—C17	0.3 (14)
O1—U—N3—C11	146.8 (6)	U—O2—C17—C16	142.7 (6)
O5—U—N3—C11	−4.6 (9)	U—O2—C17—C12	−39.3 (11)
N1—U—N3—C11	172.0 (7)	C15—C16—C17—O2	177.2 (8)
O3—U—N3—C8	77.9 (6)	C15—C16—C17—C12	−0.9 (12)
O4—U—N3—C8	−102.0 (5)	C13—C12—C17—O2	−176.6 (8)
O2—U—N3—C8	164.2 (6)	C11—C12—C17—O2	0.1 (12)
O1—U—N3—C8	−22.8 (6)	C13—C12—C17—C16	1.5 (12)
O5—U—N3—C8	−174.2 (5)	C11—C12—C17—C16	178.2 (7)
N1—U—N3—C8	2.4 (5)	U—O5—C19—C20	89.9 (15)
U—O1—C1—C2	136.4 (7)	U—O5—C19—C18	−37.1 (16)
U—O1—C1—C6	−46.3 (10)	O5—C19—C20—C21	51 (2)
O1—C1—C2—C3	176.2 (8)	C18—C19—C20—C21	174.6 (17)
C6—C1—C2—C3	−1.1 (12)	C18'—C19'—C20'—C21'	−177 (3)

### Hydrogen-bond geometry (Å, º)

**Table:**

*D*—H···*A*	*D*—H	H···*A*	*D*···*A*	*D*—H···*A*
O5—H5*O*···O1^i^	0.84	1.88	2.667 (8)	155

Symmetry code: (i) −*x*+1, −*y*+1, −*z*+1.
